# 1-(4,4′′-Difluoro-5′-meth­oxy-1,1′:3′,1′′-terphenyl-4′-yl)ethanone

**DOI:** 10.1107/S1600536811053037

**Published:** 2011-12-17

**Authors:** Hoong-Kun Fun, Madhukar Hemamalini, S. Samshuddin, B. Narayana, B. K. Sarojini

**Affiliations:** a X-ray Crystallography Unit, School of Physics, Universiti Sains Malaysia, 11800 USM, Penang, Malaysia; b Department of Studies in Chemistry, Mangalore University, Mangalagangotri 574 199, India; c Department of Chemistry, P. A. College of Engineering, Nadupadavu, Mangalore 574 153, India

## Abstract

In the title compound, C_21_H_16_F_2_O_2_, the central benzene ring is inclined at dihedral angles of 30.91 (8) and 46.88 (7)° to the two terminal fluoro-substituted rings. The dihedral angle between the two terminal fluoro-subsituted rings is 68.34 (8)°. An intra­molecular C—H⋯O hydrogen bond generates an *S*(6) ring motif. The crystal structure is stabilized by weak C—H⋯π inter­actions.

## Related literature

For a related structure and background to terphenyls, see: Fun, Chia *et al.* (2011 ▶); Samshuddin *et al.* (2011 ▶). For chalcone derivatives of the title compound, see: Fun, Hemamalini *et al.* (2011 ▶); Betz *et al.* (2011**a* ▶,b* ▶). For the synthetic procedure, see: Kotnis (1990 ▶). For hydrogen-bond motifs, see: Bernstein *et al.* (1995 ▶).
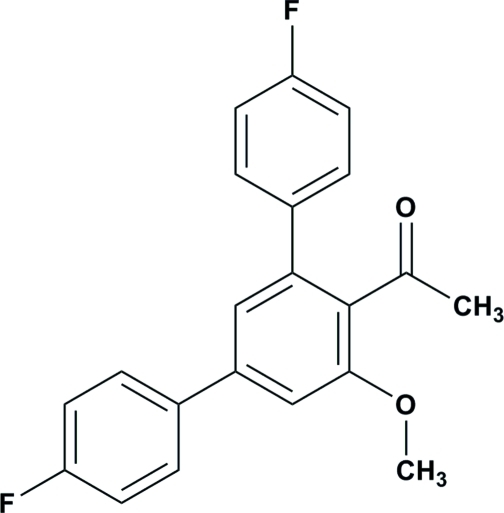


## Experimental

### 

#### Crystal data

C_21_H_16_F_2_O_2_
*M* *_r_* = 338.34
Monoclinic, 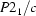

*a* = 6.0816 (7) Å
*b* = 25.997 (3) Å
*c* = 10.9061 (12) Å
β = 100.866 (2)°
*V* = 1693.4 (3) Å^3^
*Z* = 4
Mo *K*α radiation
μ = 0.10 mm^−1^
*T* = 296 K
0.74 × 0.31 × 0.10 mm

#### Data collection

Bruker APEXII DUO CCD area-detector diffractometer
Absorption correction: multi-scan (*SADABS*; Bruker, 2009 ▶) *T*_min_ = 0.930, *T*_max_ = 0.990
17519 measured reflections
4923 independent reflections
2883 reflections with *I* > 2σ(*I*)
*R*_int_ = 0.033

#### Refinement

*R*[*F* ^2^ > 2σ(*F* ^2^)] = 0.050
*wR*(*F* ^2^) = 0.167
*S* = 1.03
4923 reflections
228 parameters
H-atom parameters constrained
Δρ_max_ = 0.23 e Å^−3^
Δρ_min_ = −0.27 e Å^−3^

### 

Data collection: *APEX2* (Bruker, 2009 ▶); cell refinement: *SAINT* (Bruker, 2009 ▶); data reduction: *SAINT*; program(s) used to solve structure: *SHELXTL* (Sheldrick, 2008 ▶); program(s) used to refine structure: *SHELXTL*; molecular graphics: *SHELXTL*; software used to prepare material for publication: *SHELXTL* and *PLATON* (Spek, 2009 ▶).

## Supplementary Material

Crystal structure: contains datablock(s) global, I. DOI: 10.1107/S1600536811053037/rz2681sup1.cif

Structure factors: contains datablock(s) I. DOI: 10.1107/S1600536811053037/rz2681Isup2.hkl

Supplementary material file. DOI: 10.1107/S1600536811053037/rz2681Isup3.cml

Additional supplementary materials:  crystallographic information; 3D view; checkCIF report

## Figures and Tables

**Table 1:** Hydrogen-bond geometry (Å, °) *Cg*1 is the centroid of the C1–C6 ring.

*D*—H⋯*A*	*D*—H	H⋯*A*	*D*⋯*A*	*D*—H⋯*A*
C14—H14*A*⋯O2	0.93	2.58	3.188 (2)	124
C19—H19*A*⋯*Cg*1^i^	0.96	2.80	3.6432 (19)	147

Symmetry code: (i) 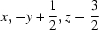.

**Fig. 1.**
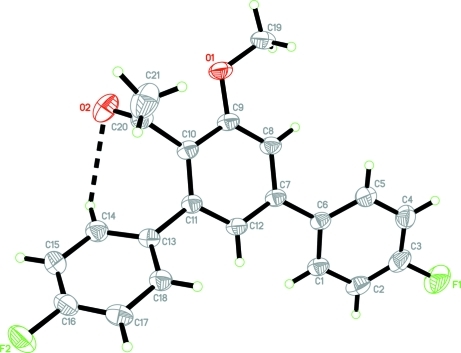
An ORTEP view of the title compound, showing 30% probability displacement ellipsoids. Intramolecular hydrogen bond is shown by a dashed line.

**Fig. 2.**
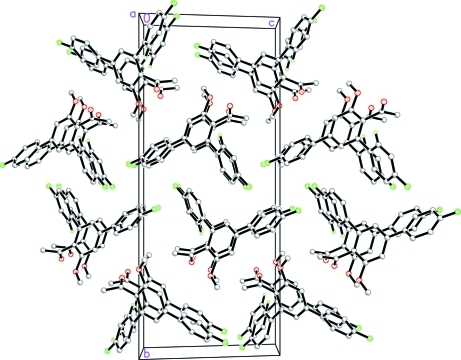
The crystal packing of the title compound viewed along the a axis.

## supplementary crystallographic information

### Comment

As a part of our ongoing studies on synthesis of terphenyl moiety from 4,4'-difluoro chalcone (Fun, Chia *et al.*, 2011; Samshuddin *et al.*, 2011), the title compound was prepared and its crystal structure is reported. We have used this acetyl terphenyl as a starting material for many chalcones (Fun, Hemamalini *et al.*, 2011; Betz *et al.*, 2011**a*,b*).

The asymmetric unit of the title compound is shown in Fig. 1.  The central benzene (C7–C12) ring is inclined at dihedral angles of 30.91 (8) and 46.88 (7)° to the two terminal fluoro-substituted phenyl (C1–C6, C13–C18) rings, respectively. The corresponding angle between the two terminal fluoro-subsituted phenyl rings is 68.34 (8)°.

An intramolecular C—H···O hydrogen bond generates an *S*(6) (Bernstein *et al.*, 1995) ring motif in the molecule (Fig. 1; Table 1). The crystal structure (Fig. 2) is stabilized by weak C—H···π interactions (Table 1) involving the C1–C6 ring (centroid Cg1).

### Experimental

The title compound was prepared by the aromatization of the cyclohexenone derivative, (6*Z*)-3,5-bis(4-fluorophenyl)-6-(1-hydroxyethylidene)cyclo hex-2-en-1-one, using iodine and methanol at reflux condition (Kotnis, 1990). Single crystal of the product was grown from a mixture of ethanol and DMF (1:1 *v*/*v*) by slow evaporation method (m.p. 391K).

### Refinement

All hydrogen atoms were positioned geometrically [C–H = 0.93 or 0.96 Å] and were refined using a riding model, with *U*_iso_(H) = 1.2 or 1.5 *U*_eq_(C). A rotating group model was applied to the methyl groups.

### Crystal data

**Table:**

C_21_H_16_F_2_O_2_	*F*(000) = 704
*M**_r_* = 338.34	*D*_x_ = 1.327 Mg m^−^^3^
Monoclinic, *P*2_1_/*c*	Mo *K*α radiation, λ = 0.71073 Å
Hall symbol: -P 2ybc	Cell parameters from 3484 reflections
*a* = 6.0816 (7) Å	θ = 2.5–24.5°
*b* = 25.997 (3) Å	µ = 0.10 mm^−^^1^
*c* = 10.9061 (12) Å	*T* = 296 K
β = 100.866 (2)°	Plate, colourless
*V* = 1693.4 (3) Å^3^	0.74 × 0.31 × 0.10 mm
*Z* = 4	

### Data collection

**Table:**

Bruker APEXII DUO CCD area-detector diffractometer	4923 independent reflections
Radiation source: fine-focus sealed tube	2883 reflections with *I* > 2σ(*I*)
graphite	*R*_int_ = 0.033
φ and ω scans	θ_max_ = 30.1°, θ_min_ = 1.6°
Absorption correction: multi-scan (*SADABS*; Bruker, 2009)	*h* = −8→8
*T*_min_ = 0.930, *T*_max_ = 0.990	*k* = −33→36
17519 measured reflections	*l* = −15→15

### Refinement

**Table:**

Refinement on *F*^2^	Primary atom site location: structure-invariant direct methods
Least-squares matrix: full	Secondary atom site location: difference Fourier map
*R*[*F*^2^ > 2σ(*F*^2^)] = 0.050	Hydrogen site location: inferred from neighbouring sites
*wR*(*F*^2^) = 0.167	H-atom parameters constrained
*S* = 1.03	*w* = 1/[σ^2^(*F*_o_^2^) + (0.0857*P*)^2^ + 0.0565*P*] where *P* = (*F*_o_^2^ + 2*F*_c_^2^)/3
4923 reflections	(Δ/σ)_max_ = 0.001
228 parameters	Δρ_max_ = 0.23 e Å^−^^3^
0 restraints	Δρ_min_ = −0.27 e Å^−^^3^

### Special details

**Table:**

Geometry. All s.u.'s (except the s.u. in the dihedral angle between two l.s. planes) are estimated using the full covariance matrix. The cell s.u.'s are taken into account individually in the estimation of s.u.'s in distances, angles and torsion angles; correlations between s.u.'s in cell parameters are only used when they are defined by crystal symmetry. An approximate (isotropic) treatment of cell s.u.'s is used for estimating s.u.'s involving l.s. planes.
Refinement. Refinement of F^2^ against ALL reflections. The weighted R-factor wR and goodness of fit S are based on F^2^, conventional R-factors R are based on F, with F set to zero for negative F^2^. The threshold expression of F^2^ > 2σ(F^2^) is used only for calculating R-factors(gt) etc. and is not relevant to the choice of reflections for refinement. R-factors based on F^2^ are statistically about twice as large as those based on F, and R- factors based on ALL data will be even larger.

### Fractional atomic coordinates and isotropic or equivalent isotropic displacement parameters (Å^2^)

**Table:**

	*x*	*y*	*z*	*U*_iso_*/*U*_eq_	
F1	0.7590 (3)	0.93216 (6)	0.59950 (13)	0.1136 (5)	
F2	−0.8096 (2)	1.00075 (4)	−0.25890 (13)	0.0932 (4)	
O1	0.0345 (2)	0.73907 (4)	0.00260 (11)	0.0617 (3)	
O2	−0.4540 (2)	0.77193 (5)	−0.13608 (13)	0.0825 (4)	
C1	0.2502 (3)	0.91677 (6)	0.37217 (14)	0.0527 (4)	
H1A	0.1061	0.9302	0.3545	0.063*	
C2	0.3999 (3)	0.93447 (7)	0.47550 (16)	0.0647 (5)	
H2A	0.3569	0.9592	0.5279	0.078*	
C3	0.6109 (4)	0.91488 (7)	0.49859 (17)	0.0705 (5)	
C4	0.6816 (3)	0.87831 (8)	0.42537 (18)	0.0711 (5)	
H4A	0.8267	0.8655	0.4444	0.085*	
C5	0.5325 (3)	0.86066 (6)	0.32199 (15)	0.0586 (4)	
H5A	0.5795	0.8362	0.2703	0.070*	
C6	0.3128 (3)	0.87898 (5)	0.29407 (13)	0.0456 (3)	
C7	0.1516 (2)	0.85840 (5)	0.18611 (13)	0.0457 (3)	
C8	0.1709 (3)	0.80732 (6)	0.14915 (13)	0.0502 (4)	
H8A	0.2819	0.7863	0.1935	0.060*	
C9	0.0255 (3)	0.78806 (5)	0.04698 (14)	0.0493 (3)	
C10	−0.1469 (3)	0.81858 (5)	−0.01979 (13)	0.0467 (3)	
C11	−0.1702 (2)	0.86946 (5)	0.01721 (13)	0.0455 (3)	
C12	−0.0200 (2)	0.88852 (5)	0.12041 (13)	0.0465 (3)	
H12A	−0.0354	0.9223	0.1457	0.056*	
C13	−0.3440 (2)	0.90439 (5)	−0.05353 (12)	0.0454 (3)	
C14	−0.5668 (3)	0.88925 (6)	−0.08926 (16)	0.0573 (4)	
H14A	−0.6106	0.8570	−0.0661	0.069*	
C15	−0.7240 (3)	0.92160 (7)	−0.15882 (17)	0.0660 (5)	
H15A	−0.8721	0.9112	−0.1837	0.079*	
C16	−0.6553 (3)	0.96922 (7)	−0.18989 (15)	0.0612 (4)	
C17	−0.4413 (3)	0.98642 (6)	−0.15416 (16)	0.0610 (4)	
H17A	−0.4010	1.0194	−0.1748	0.073*	
C18	−0.2853 (3)	0.95354 (6)	−0.08626 (14)	0.0525 (4)	
H18A	−0.1380	0.9645	−0.0619	0.063*	
C19	0.2322 (3)	0.70987 (6)	0.04690 (18)	0.0668 (5)	
H19A	0.2267	0.6783	0.0007	0.100*	
H19B	0.2417	0.7023	0.1339	0.100*	
H19C	0.3612	0.7293	0.0359	0.100*	
C20	−0.2837 (3)	0.79540 (6)	−0.13640 (15)	0.0548 (4)	
C21	−0.1930 (4)	0.80270 (11)	−0.25154 (18)	0.0997 (8)	
H21A	−0.2653	0.7793	−0.3146	0.150*	
H21B	−0.0348	0.7961	−0.2344	0.150*	
H21C	−0.2198	0.8374	−0.2805	0.150*	

### Atomic displacement parameters (Å^2^)

**Table:**

	*U*^11^	*U*^22^	*U*^33^	*U*^12^	*U*^13^	*U*^23^
F1	0.1147 (10)	0.1119 (10)	0.0921 (9)	−0.0152 (8)	−0.0369 (8)	−0.0252 (8)
F2	0.0933 (8)	0.0800 (8)	0.0952 (9)	0.0405 (6)	−0.0111 (7)	0.0045 (6)
O1	0.0756 (8)	0.0373 (6)	0.0671 (7)	0.0058 (5)	0.0004 (6)	−0.0108 (5)
O2	0.0853 (9)	0.0775 (9)	0.0815 (9)	−0.0340 (7)	0.0074 (7)	−0.0082 (7)
C1	0.0663 (9)	0.0407 (8)	0.0495 (8)	−0.0009 (7)	0.0069 (7)	0.0013 (6)
C2	0.0898 (13)	0.0491 (9)	0.0520 (9)	−0.0063 (9)	0.0056 (9)	−0.0073 (7)
C3	0.0818 (12)	0.0611 (11)	0.0591 (10)	−0.0144 (9)	−0.0106 (9)	−0.0027 (8)
C4	0.0575 (10)	0.0697 (12)	0.0788 (12)	−0.0044 (8)	−0.0055 (9)	0.0030 (10)
C5	0.0603 (9)	0.0524 (9)	0.0618 (9)	−0.0009 (7)	0.0084 (8)	−0.0030 (7)
C6	0.0576 (8)	0.0351 (7)	0.0433 (7)	−0.0028 (6)	0.0072 (6)	0.0044 (5)
C7	0.0572 (8)	0.0358 (7)	0.0439 (7)	−0.0007 (6)	0.0092 (6)	0.0012 (5)
C8	0.0618 (9)	0.0367 (7)	0.0497 (8)	0.0041 (6)	0.0046 (7)	0.0008 (6)
C9	0.0628 (9)	0.0340 (7)	0.0509 (8)	−0.0001 (6)	0.0101 (7)	−0.0020 (6)
C10	0.0559 (8)	0.0383 (7)	0.0458 (7)	−0.0034 (6)	0.0096 (6)	−0.0011 (6)
C11	0.0513 (8)	0.0387 (7)	0.0465 (7)	−0.0005 (6)	0.0095 (6)	0.0010 (6)
C12	0.0578 (8)	0.0349 (7)	0.0461 (7)	0.0025 (6)	0.0079 (6)	−0.0026 (5)
C13	0.0528 (8)	0.0404 (7)	0.0440 (7)	0.0030 (6)	0.0118 (6)	−0.0010 (6)
C14	0.0554 (9)	0.0489 (9)	0.0683 (10)	−0.0005 (7)	0.0137 (8)	0.0020 (7)
C15	0.0510 (9)	0.0665 (12)	0.0785 (11)	0.0080 (8)	0.0067 (8)	−0.0084 (9)
C16	0.0680 (10)	0.0585 (10)	0.0543 (9)	0.0224 (8)	0.0044 (8)	−0.0016 (8)
C17	0.0752 (11)	0.0441 (9)	0.0651 (10)	0.0090 (8)	0.0171 (9)	0.0089 (7)
C18	0.0545 (8)	0.0444 (8)	0.0586 (9)	0.0012 (6)	0.0106 (7)	0.0015 (7)
C19	0.0764 (11)	0.0456 (9)	0.0781 (12)	0.0123 (8)	0.0141 (9)	−0.0103 (8)
C20	0.0607 (9)	0.0445 (8)	0.0571 (9)	−0.0012 (7)	0.0057 (7)	−0.0054 (7)
C21	0.0960 (16)	0.145 (2)	0.0591 (11)	−0.0280 (15)	0.0183 (11)	−0.0237 (13)

### Geometric parameters (Å, °)

**Table:**

F1—C3	1.360 (2)	C10—C20	1.508 (2)
F2—C16	1.3610 (19)	C11—C12	1.3997 (19)
O1—C9	1.3671 (17)	C11—C13	1.4930 (19)
O1—C19	1.427 (2)	C12—H12A	0.9300
O2—C20	1.203 (2)	C13—C18	1.391 (2)
C1—C2	1.387 (2)	C13—C14	1.394 (2)
C1—C6	1.399 (2)	C14—C15	1.387 (2)
C1—H1A	0.9300	C14—H14A	0.9300
C2—C3	1.359 (3)	C15—C16	1.369 (3)
C2—H2A	0.9300	C15—H15A	0.9300
C3—C4	1.362 (3)	C16—C17	1.362 (3)
C4—C5	1.385 (2)	C17—C18	1.383 (2)
C4—H4A	0.9300	C17—H17A	0.9300
C5—C6	1.397 (2)	C18—H18A	0.9300
C5—H5A	0.9300	C19—H19A	0.9600
C6—C7	1.483 (2)	C19—H19B	0.9600
C7—C12	1.3904 (19)	C19—H19C	0.9600
C7—C8	1.399 (2)	C20—C21	1.476 (3)
C8—C9	1.379 (2)	C21—H21A	0.9600
C8—H8A	0.9300	C21—H21B	0.9600
C9—C10	1.404 (2)	C21—H21C	0.9600
C10—C11	1.398 (2)		
			
C9—O1—C19	117.60 (12)	C7—C12—H12A	119.1
C2—C1—C6	121.04 (16)	C11—C12—H12A	119.1
C2—C1—H1A	119.5	C18—C13—C14	118.05 (14)
C6—C1—H1A	119.5	C18—C13—C11	120.02 (13)
C3—C2—C1	118.57 (17)	C14—C13—C11	121.93 (13)
C3—C2—H2A	120.7	C15—C14—C13	121.03 (16)
C1—C2—H2A	120.7	C15—C14—H14A	119.5
C2—C3—F1	118.96 (19)	C13—C14—H14A	119.5
C2—C3—C4	122.96 (16)	C16—C15—C14	118.18 (16)
F1—C3—C4	118.09 (19)	C16—C15—H15A	120.9
C3—C4—C5	118.56 (18)	C14—C15—H15A	120.9
C3—C4—H4A	120.7	F2—C16—C17	118.85 (16)
C5—C4—H4A	120.7	F2—C16—C15	118.08 (17)
C4—C5—C6	121.13 (16)	C17—C16—C15	123.08 (15)
C4—C5—H5A	119.4	C16—C17—C18	118.17 (16)
C6—C5—H5A	119.4	C16—C17—H17A	120.9
C5—C6—C1	117.73 (14)	C18—C17—H17A	120.9
C5—C6—C7	120.81 (13)	C17—C18—C13	121.45 (15)
C1—C6—C7	121.46 (14)	C17—C18—H18A	119.3
C12—C7—C8	118.78 (13)	C13—C18—H18A	119.3
C12—C7—C6	121.72 (12)	O1—C19—H19A	109.5
C8—C7—C6	119.49 (13)	O1—C19—H19B	109.5
C9—C8—C7	120.08 (14)	H19A—C19—H19B	109.5
C9—C8—H8A	120.0	O1—C19—H19C	109.5
C7—C8—H8A	120.0	H19A—C19—H19C	109.5
O1—C9—C8	124.16 (14)	H19B—C19—H19C	109.5
O1—C9—C10	114.76 (13)	O2—C20—C21	121.87 (16)
C8—C9—C10	121.08 (13)	O2—C20—C10	122.61 (15)
C11—C10—C9	119.45 (13)	C21—C20—C10	115.52 (15)
C11—C10—C20	123.47 (13)	C20—C21—H21A	109.5
C9—C10—C20	116.81 (12)	C20—C21—H21B	109.5
C10—C11—C12	118.71 (13)	H21A—C21—H21B	109.5
C10—C11—C13	121.79 (13)	C20—C21—H21C	109.5
C12—C11—C13	119.44 (12)	H21A—C21—H21C	109.5
C7—C12—C11	121.86 (13)	H21B—C21—H21C	109.5
			
C6—C1—C2—C3	1.0 (3)	C20—C10—C11—C12	−173.84 (14)
C1—C2—C3—F1	179.78 (16)	C9—C10—C11—C13	177.29 (14)
C1—C2—C3—C4	−0.5 (3)	C20—C10—C11—C13	3.5 (2)
C2—C3—C4—C5	0.7 (3)	C8—C7—C12—C11	−1.3 (2)
F1—C3—C4—C5	−179.64 (17)	C6—C7—C12—C11	178.96 (14)
C3—C4—C5—C6	−1.3 (3)	C10—C11—C12—C7	0.4 (2)
C4—C5—C6—C1	1.7 (2)	C13—C11—C12—C7	−176.95 (13)
C4—C5—C6—C7	−177.57 (15)	C10—C11—C13—C18	−131.38 (16)
C2—C1—C6—C5	−1.6 (2)	C12—C11—C13—C18	45.89 (19)
C2—C1—C6—C7	177.70 (14)	C10—C11—C13—C14	48.3 (2)
C5—C6—C7—C12	−149.43 (15)	C12—C11—C13—C14	−134.44 (16)
C1—C6—C7—C12	31.3 (2)	C18—C13—C14—C15	2.0 (2)
C5—C6—C7—C8	30.9 (2)	C11—C13—C14—C15	−177.66 (14)
C1—C6—C7—C8	−148.36 (15)	C13—C14—C15—C16	−1.0 (3)
C12—C7—C8—C9	1.9 (2)	C14—C15—C16—F2	179.55 (15)
C6—C7—C8—C9	−178.40 (14)	C14—C15—C16—C17	−1.0 (3)
C19—O1—C9—C8	−14.0 (2)	F2—C16—C17—C18	−178.69 (14)
C19—O1—C9—C10	166.16 (14)	C15—C16—C17—C18	1.8 (3)
C7—C8—C9—O1	178.61 (14)	C16—C17—C18—C13	−0.7 (2)
C7—C8—C9—C10	−1.5 (2)	C14—C13—C18—C17	−1.1 (2)
O1—C9—C10—C11	−179.55 (13)	C11—C13—C18—C17	178.55 (14)
C8—C9—C10—C11	0.6 (2)	C11—C10—C20—O2	−93.9 (2)
O1—C9—C10—C20	−5.3 (2)	C9—C10—C20—O2	92.1 (2)
C8—C9—C10—C20	174.82 (15)	C11—C10—C20—C21	87.0 (2)
C9—C10—C11—C12	0.0 (2)	C9—C10—C20—C21	−87.0 (2)

### Hydrogen-bond geometry (Å, °)

**Table:**

Cg1 is the centroid of the C1–C6 ring.

**Table:**

*D*—H···*A*	*D*—H	H···*A*	*D*···*A*	*D*—H···*A*
C14—H14A···O2	0.93	2.58	3.188 (2)	124.
C19—H19A···Cg1^i^	0.96	2.80	3.6432 (19)	147.

Symmetry codes: (i) *x*, −*y*+1/2, *z*−3/2.
